# Dose escalation with stereotactic body radiation therapy boost for locally advanced non small cell lung cancer

**DOI:** 10.1186/1748-717X-8-179

**Published:** 2013-07-10

**Authors:** Sana D Karam, Zachary D Horne, Robert L Hong, Don McRae, David Duhamel, Nadim M Nasr

**Affiliations:** 1 Department of Radiation Oncology, Georgetown University Hospital, 3800 Reservoir Road, NW, Washington, DC 20007, USA; 2 Department of Radiation Oncology, Virginia Hospital Center, Arlington, VA, USA; 3 Department of Pulmonary/Critical Care Medicine, Virginia Hospital Center, Arlington, VA, USA

**Keywords:** SBRT, SABR, Cyberknife, Boost, Dose escalation, Locally advanced, Stage IIIA, Stage IIIB, Nodal, Age

## Abstract

**Introduction:**

Low survival outcomes have been reported for the treatment of locally advanced non small cell lung cancer (LA-NSCLC) with the standard of care treatment of concurrent chemoradiation (cCRT). We present our experience of dose escalation using stereotactic body radiosurgery (SBRT) following conventional cCRT for patients with LA-NSCLC.

**Methods:**

Sixteen patients with a median age of 67.5 treated with fractionated SBRT from 2010 to 2012 were retrospectively analyzed. Nine (56%) of the patients had stage IIIB, 6 (38%) has stage IIIA, and 1 (6%) had recurrent disease. Majority of the patients (63%) presented with N2 disease. All patients had a PET CT for treatment planning. Patients received conventional cCRT to a median dose of 50.40 Gy (range 45–60) followed by an SBRT boost with an average dose of 25 Gy (range 20–30) given over 5 fractions.

**Results:**

With a median follow-up of 14 months (range, 1–14 months), 1-year overall survival (OS), progression free survival (PFS), local control (LC), regional control (RC), and distant control (DC) rates were, 78%, 42%, 76%, 79%, and 71%, respectively. Median times to disease progression and regional failure were 10 months and 18 months, respectively. On univariate analysis, advanced age and nodal status were worse prognostic factors of PFS (p < 0.05). Four patients developed radiation pneumonitis and one developed hemoptysis. Treatment was interrupted in one patient who required hospitalization due to arrhythmias and pneumonia.

**Conclusion:**

Risk adaptive dose escalation with SBRT following external beam radiotherapy is possible and generally tolerated treatment option for patients with LA-NSCLC.

## Introduction

For patients with locally advanced lung cancer concurrent chemoradiation (cCRT) provides improved survival and local control compared to sequential chemotherapy and radiation but with higher toxicity [1,2]. In order to improve the therapeutic ratio, newer radiosensitizing chemotherapeutic agents were introduced [3]. However, when survival and pattern of failure following cCRT for locally advanced NSCLC with conventional and new chemotherapy agents were compared, no change in survival or failure pattern was observed in a recent system review of the literature [3]. Of the 15 studies reviewed in the analysis, only one study varied in its radiation therapy technique and dosing leading to the conclusion that local control cannot be reliably achieved with conventional radiation dosing and techniques. Local control also has an impact on distant metastases [4]. As most local recurrences after cCRT for locally advanced NSCLC occur in the area of high radiation dose within the tumor [5], radiation dose escalation by 10 Gy to maximal dose of 74 Gy has been shown to reduce local recurrence rate 15% [6,7].

However, the recently presented RTOG 0617 trial, comparing 74 Gy in 37 fractions with 60 Gy in 30 fractions, did not show an OS benefit for the higher dose [8]. This was a 2 × 2 factorial design study designed to test these radiation doses with carboplatin and paclitaxel, with or without cetuximab, for Stage III NSCLC. A planned interim analysis led to the closure of the high dose arms since the predetermined futility threshold was crossed, indicating a low likelihood of a survival benefit to high dose RT as used in this trial with additional accrual and follow up. Both standard and high dose RT were delivered with standard fractionation (2 Gy per fraction), resulting in longer overall treatment time for the high dose arm [8]. This suggests that unless radiation dose escalation can be safely delivered in clinical trials, there will be little improvement in patient outcome.

The introduction of stereotactic body radiotherapy (SBRT) provides a feasible technique of radiation which effectively increased tumor dose while sparing normal tissues. In early stages NSCLC, local control and survival were comparable to surgery in patients with multiple co-mor*bid*ities precluding surgery [9]. SBRT may allow increased local control and survival for locally advanced NSCLC as well by targeting the PET positive tumor and grossly enlarged mediastinal lymph nodes to a high radiation dose [9]. In this manuscript we review our experience using SBRT boost as a means for dose escalation for patients with locally advanced NSCLC.

## Methods

### Eligibility

After approval by the Virginia Hospital Center institutional review board was obtained, all patients with a diagnosis of clinically staged primary IIIA-IV NSCLC or recurrent stage IA/IB NSCLC (per the American Joint Committee on Cancer staging manual, 7th edition) treated between 2010 and 2012 with either definitive or palliative intent radiotherapy at our institution were identified. Patients who had received conventionally fractionated IMRT followed by a hypofractionated radiotherapy boost to residual disease were retrospectively reviewed. All patients had a histologically confirmed diagnosis of NSCLC, and all had available a history; a physical examination; a Positron Emission Tomography computed tomography (PET/CT) imaging of the chest, abdomen, and pelvis; and brain magnetic resonance imaging (MRI). All patients were determined by thoracic surgeons to be inoperable. Patients who had had prior thoracic radiotherapy were excluded. Charts were reviewed to determine patterns of disease failure, toxicity (as defined by the *Common Terminology Criteria for Adverse Events,* version 3.0), and outcome.

### IMRT treatment planning

For simulation, each patient was placed in the treatment position on a flat table to ensure reproducibility of setup during treatment and an FDG-PET/CT scan was obtained. In patients without contraindication, IV contrast was utilized to delineate major blood vessels. Target volume definitions were made in accordance with the 1993 ICRU Report #62.

The gross tumor volume (GTV) was defined as the primary tumor and clinically positive lymph nodes seen on the planning CT or PET scan (SUV >3) and generally drawn on lung windows. The clinical tumor volume (CTV) was defined as the GTV expanded by a 0.5-1.0 cm margin as appropriate. To form the planning tumor volume (PTV), at least 1.0 cm was added in the superior-inferior direction and 0.5 cm in the axial plane with an additional setup margin of 0.5 cm. Thus, the total PTV included the CTV plus a total margin of at least 1.5 cm to the superior-inferior dimension and at least 1.0 cm in the axial plane. Normal anatomy identified on each CT planning image included the right and left lungs (separately), heart, skin, esophagus, and spinal cord.

### IMRT dosimetry

All plans were created using Eclipse utilizing beams of [6-18] MV photons, taking into account the inhomogeneity of lung tissue. Normalization of the treatment plan covered 95% of the PTV with the prescription dose such that the minimum PTV dose did not fall below 95% of the prescription. To ensure good coverage, ≥ 99% of the PTV received ≥ 93% of the prescribed dose. No more than 1 cm^3^ of tissue outside the PTV received ≥ 120% of the prescribed dose. For critical structures, dosing guidelines from QUANTEC were followed [10].

### Treatment delivery

Radiation therapy (RT) commenced on day 1 of chemotherapy. Patients were treated 5 days per week with routine position verification using KV imaging. On days when chemotherapy was given concurrently with RT, attempts were made to administer chemotherapy prior to RT. Need for treatment breaks were minimized through nutrition consults, pain control, and medical management.

### Fiducial placement

In preparation for the stereotactic radiosurgery boost to residual disease, most patients received fiducial placement for real time image guidance (Conventional gold seeds, Best Medical International, Springfield, VA), which were placed into adjacent soft tissue either brochcoscopically, or percutaneously. One to four gold fiducials 0.8–1 mm in diameter by 3–7 mm in length are usually placed non-colinearly for best translational correction of the radiation beam pointing. To minimize fiducial migration a 1–2 week period of time was allowed before simulation was completed in order to decrease procedural edema and permit fibrosis and fixation of fiducials. To address concerns of accelerated repopulation, minimization of time between IMRT and CyberKnife™ boost was ideally achieved in patients by placing the fiducials sometime in the last two weeks of their IMRT treatment, allowing them to be simulated near the end of IMRT and beginning SRS within just a few days of the conclusion of IMRT.

For lesions which were adjacent to, and do not move independent of the spine, spinal tracking which uses spine bony landmarks (X-Sight Spine™) was utilized instead. Pretreatment digitally reconstructed radiographs (DRRs) were generated from CT scans. Three-dimensional target displacements and global rotations of spinal structures were determined by comparing radiographs with DRRs. Translations and global rotations were aligned during patient setup and corrected during treatment delivery.

### Radiosurgery treatment planning

Planning for the radiosurgery boost began one to two weeks after fiducial placement. A new fine cut (1.25 mm) CT was used for targeting and treatment planning. The planning CT scans were done with IV contrast as needed to allow better distinction between tumor and adjacent vessels or atelectasis. The residual disease, comprising the primary tumor and involved nodes, was outlined by a radiation oncologist and designated as gross tumor volume (GTV). The target was generally drawn using CT pulmonary windows. However, soft tissue window with contrast was sometimes used to avoid inclusion of adjacent vessels, atelectasis, or mediastinal or chest wall structures within the GTV. This target included only abnormal CT signal consistent with gross tumor and/or nodal volume (i.e., the GTV and the clinical target volume (CTV) were identical). The pre-treatment PET/CT was fused on all patients in order to assist in defining the GTV. Additional margin of 5 mm was added to the GTV to constitute the PTV, but adjustments were made by the treating physician based on tumor location, proximity of critical structures, and tumor motion during treatment.

### Radiosurgery dosimetry

Three-dimensional non-coplanar beam arrangements were custom designed for each case to deliver highly conformal prescription dose distributions. Generally, more beams were used for larger lesions. As such, prescription lines covering the PTV were typically around 80% but ranging between 60-90% line rather than the more traditional 95-100%. Higher isodoses (hotspots) were manipulated to occur within the target and not in adjacent normal tissue.

Treatment conformality was determined in all patient using the new conformity index (NCI), which was calculated by the formula: Treatment Volume × Prescription Isodose Line/(Volume of Target Covered by Prescription Isodose Line)^2^. The following critical structures were contoured: Spinal Cord, esophagus, brachial plexus, heart, trachea and proximal bronchial tree, proximal trachea, whole lung, and skin. As a general rule, prescription doses were dictated by tolerance of surrounding structures, which were in accordance with the AAPM Task Group 101 [11]. In other words, risk adaptive radiotherapy was used whereby selectively boosting of tumor volumes was accomplished without violating normal tissue complication constraints using information from functional imaging. The boost was generally performed in one plan encompassing all targets with the exception of one case where mediastinal disease could not be encompassed by one plan without exceeding tolerance of critical structures. The mediastinal disease was therefore treated in a separate boost plan from the primary lung tumor.

BED was calculated for the IMRT and SBRT portions of the treatment using the formula BED = nd(1 + d/*α*/*β*), where n is the number of fractions and d is the dose per fraction, and using an α/β ratio of 10 for acute reacting tissues such as lung. The cumulative BED was established by combining the calculated BED of IMRT and SBRT.

### Radiosurgery treatment delivery and post-treatment follow-up

Tumors were actively tracked in real time during treatment using Synchrony™. For the treatments the patient wore a tight-fitting elastic vest. Three beacons emitting visible red light pulsed at 30 Hz were placed on this vest. The beacon positions were monitored by a camera mounted at the foot end of the patient couch. This camera continuously recorded the position of the markers during the patients’ respiration cycle. In parallel, a series of X-ray images of the internal fiducials were taken with the patient breathing freely. These images established the position of the fiducials and thus the tumor at the time of these images. The time-stamps of the beacon data (as captured by the camera) and the location data of the internal fiducials (as determined at the instant of the X-ray images) were synchronized. From a series of these images a correlation model between the external and internal positions was established. Thus, using the instantaneous information from the external beacon, the position of the tumor at that instant was calculated. Using this dynamic model, the robot was able to track the tumor motion in real time while the radiation is being delivered. As treatment commences X-ray images were taken, either before each therapeutic beam or less frequently for systematic breathers, updating the model. For follow-up, clinical examinations and imaging studies were performed at 3-month intervals from the end of the treatment. Given that SBRT-induced consolidation can be very difficult to distinguish from local recurrence using post-treatment CT based RECIST criteria, PET scan was obtained on all patients at follow-up. Residual PET uptake and maximal SUVs were used to distinguish fibrosis from residual disease [12,13].

### Statistical analysis

Progression-free survival (PFS) was defined as the time from the first day of SBRT treatment to local, regional, or distant failure or last follow-up in living patients without evidence of recurrence or progression. Local control (LC), local failure, regional failure, and distant failures were defined as previously described [14]. Briefly, local failure was defined as disease persistence or recurrence in the targeted gross tumor volume (primary tumor, involved nodes). Regional failure was defined as presence of nodal or intrathoracic disease outside of the targeted gross tumor volume. Patients were censored at the time of death. Overall survival (OS) was the time from SBRT treatment until death or last follow-up. Interpretation of available FDGPET/CT, and CT scans with correlative clinical examinations were used to assess for response of the treated lesion 3 months after SBRT. Log rank tests and Cox regression models were used to evaluate the association between clinical factors and each survival outcome. The independent variables considered were stage (recurrent, IIIA, IIIB), gender (male, female), BED (<100, >100 Gy), chemotherapy (yes, no), nodal status (<N2, ≥N2), age in years, GTV in cubic centimeters, and SBRT dose in Gy. Kaplan-Meier plots are presented for selected significant factors. Acute toxicities examined included fatigue, chest pain, shortness of breath, cough, hemoptysis, wheezing, and esophagitis that occurred during treatment or within the first two weeks following the end of treatment. Radiation pneumonitis was examined as a subacute toxicity in all patients. Analyses were performed in SAS version 9.2 (SAS Institute Inc., Cary, NC).

## Results

### Patient characteristics

A total of 16 patients with locally advanced NSCLC received SBRT boost at our institution. Baseline patient and disease characteristics are listed in Table 1. Median patient age at the time of treatment was 67.5 (range 52–90) for all patients, with 44% males and 56% females. The median KPS was 90 (range 60–100) and the histology varied between squamous (19%), adenocarcinoma (50%), and nonspecified NSCLC (31%). Six (38%) of the patients had stage IIIA, 9 (56%) has stage IIIB, and 1 (6%) had recurrent disease that had initially presented with stage I disease and treated with lobectomy but recurred with hilar and paratracheal node metastasis. Majority of the patients (63%) presented with N2 disease. The median tumor volume was approximately 66 cc (range 4.9-170) with a median maximal diameter of 5 cm (range 2.4-9.5).

**Table 1:** Patients characteristics

	
Age (median, range)	(67.5, 52–90)
KPS (median, range)	(90, 60–100)
Gender (Male, Female)	(9,7) (44%, 56%)
Histology (Adenocarcinoma, Squamous, NSCLC) (%)	(8, 3, 5) (50, 19, 31)
Clinical Stage (recurrent IA, IIIA, IIIB) (%)	(1, 6, 9) (6, 38, 56)
N Stage (0, 1, 2, 3) (%)	(4, 1, 10, 1) (25, 6, 63, 6)
Maximum Diameter in cm (mean, median, range)	(5.2, 5.0, 2.4-9.5)
Volume in cc (mean, median, range)	(74.2, 65.7, 4.9-170)

Abbreviations: *KPS* Karnofsky Performance Status, *NSCLC* Non Small Cell Lung Cancer.

### Treatment characteristics

Treatment characteristics are presented in Table 2. Patients received a median IMRT dose of 50.40 Gy (range 45–60) with a median fractionation regimen of 1.8 Gy (range 1.8-2.0) in 28 fractions (range 25–30). SBRT boost dose averaged 25 Gy (range 20–30) given over 5 fractions with a median dose of 5 Gy thus bringing the total cumulative dose to a median of 75.4 Gy (range 65-90 Gy). The cumulative BED10 ranged from 81–120 Gy with a median value of 97 Gy. On average 96% of the PTV was covered by the prescription isodose line (range 80-100%) and the median conformity index was 1.43 (range 1.23-2.10). The SBRT boost followed the IMRT treatment and was given over 5–11 days (median 7 days) with the exception of the one patient that required hospitalization after the second SBRT treatment where the duration was extended for 30 days. The median elapsed time between IMRT treatment and SBRT boost treatment was 20 days but the range varied between irradiation and 7 and 97. One patient had extended treatment time due to development of arrhythmias at the end of his IMRT requiring hospitalization while in another fiducial migration outside the tumor and requirement of fiducial replacement were the delaying factors. The majority (95%) of the patients received cCRT chemotherapy except for one patient who received chemotherapy prior to radiation due to patient’s preference.

**Table 2:** Treatment characteristics

	**Mean, Median (Range)**
Total Prescribed Dose (Gy)	76.4, 75.4 (65–90)
SBRT Prescription IDL (%)	80.8, 80.0 (70–92)
Number of Boost Fractions	5, 5, (5–5)
SBRT Dose per Fraction (Gy)	5, 5 (4–6)
Total Boost Dose	25, 25 (20–30)
Duration of SBRT Treatment	9, 7 (5–11)
Number of IMRT Fractions	27.6, 28, (25–30)
IMRT Dose per Fraction	1.87, 1.8 (1.8-2.0)
Total IMRT Dose	51.42, 50.40 (45–60)
Duration of IMRT treatment	41.5, 42 (35–49)
Duration between IMRT and SBRT	25, 20 (7–97)
Cumulative BED10	98.8, 97.0 (81.1-120)
Coverage	95.7, 96 (80–100)
NCI	1.53, 1.43 (1.23-2.10)
Chemotherapy (concurrent, sequential)	(15, 1) (94%, 6%)

Abbreviations: *IDL* Isodose Line, *BED* Biologically Effective Dose, *NCI* New Conformity Index, which is calculated by the formula: Treatment Volume × Prescription Isodose Line/(Volume of Target Covered by Prescription Isodose Line)^2^.

### Clinical outcomes and prognostic factors

**Figure 1:**
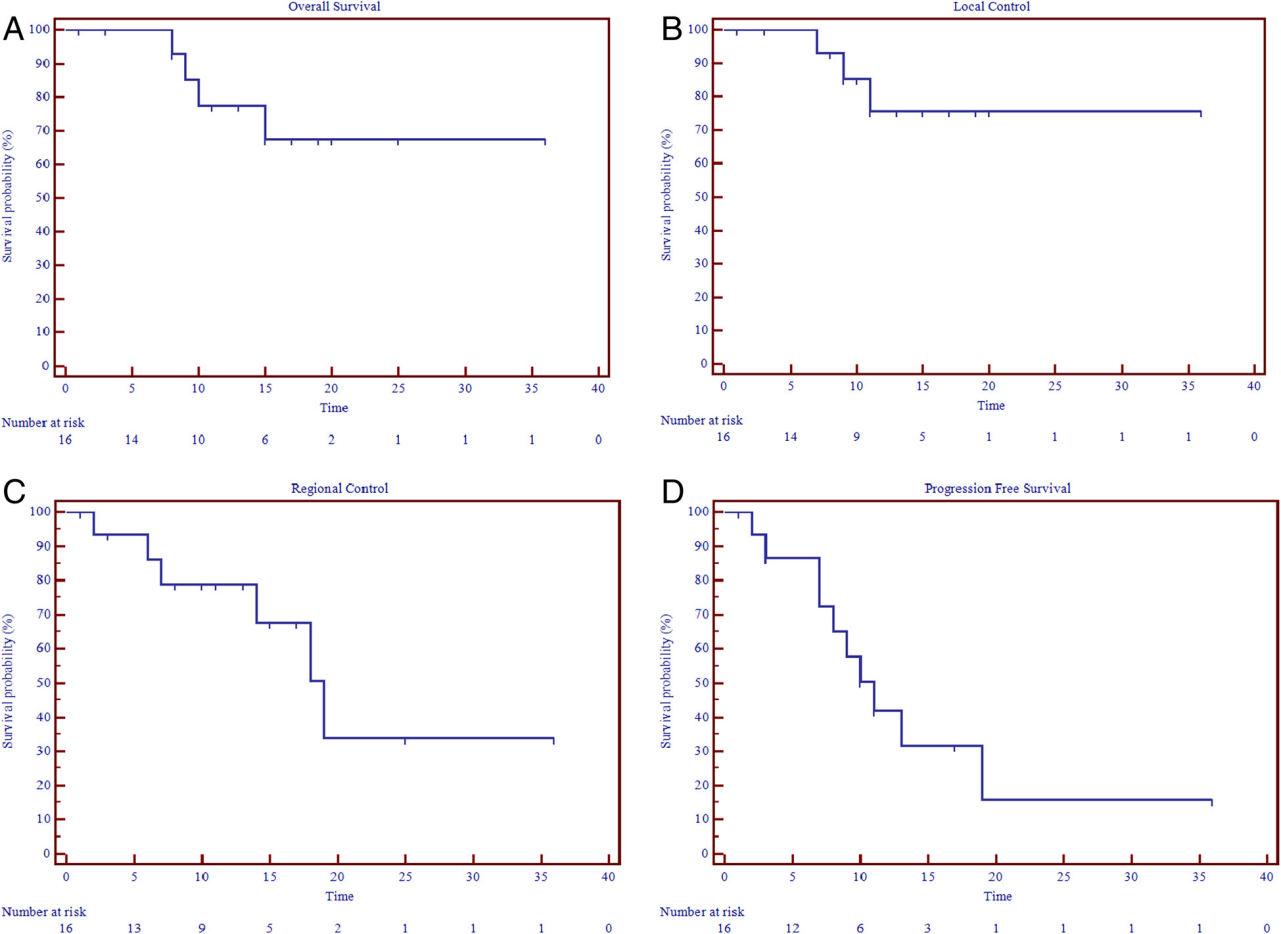
**Survival outcomes. A**. Local control. **B**. Regional Control. **C**. Progression Free Survival. **D**. Overall Survival.

**Figure 2:**
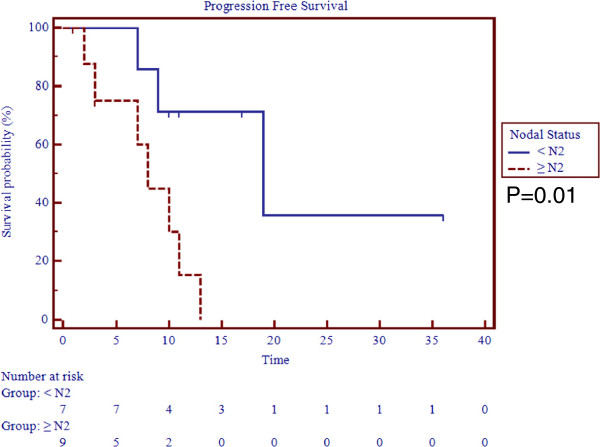
Progression free survival by nodal status.

The median followup was 14 months (0–36). The actuarial 1-year overall survival (OS), progression free survival (PFS), local control (LC), regional control (RC), and distant control (DC) rates were, 78%, 42%, 76%, 79%, and 71% (Table 3, Figure 1). At a median followup of 14 months the survival outcomes were not changed for OS, LC, but drop to 61.5%, 68%, and 32% for DC, RC, and PFS, respectively. The median time to disease progression was 10 months and median time for regional failure, which was the most prevalent pattern of failure, was 18 months. All other median times were not reached. On univariate analysis, age > 70 was a statistically significant predictor for regional failure (p = 0.03) whereas advanced age and nodal status with N ≥ 2 were worse prognostic indicators for PFS (p < 0.05) (Figure 2). No other correlates for other survival outcomes reached statistical significance.

**Table 3:** Percent actuarial survival outcomes and control rates

	
Median Followup (range in months)	14 (1–36)
Local control at 1 year, at 14 months, (failed/controlled)	76%, 76% (3/13)
Regional control at 1 year, at 14 months, (failed/controlled)	79%, 68% (6/10)
Distant control at 1 year, at 14 months, (failed/controlled)	71%, 61.5% (5/11)
PFS at 1 year, at 14 months (progression/no progression)	42%, 32% (10/6)
Overall survival at 1 year, at 14 months (dead/alive)	78%, 78%, (4/12)

### Toxicity

The most common acute toxicities were fatigue and dry cough experienced by 56% [9] and 50% [8] of the patients, respectively (Table 4). For the majority of the cases both symptoms were self limited and resolved within 6–8 weeks following termination of treatment. In one patient with stage IIIA disease treated with concurrent IMRT followed by boost for a total cumulative dose of 90 Gy, his cough dry cough progressed into hemoptysis and pneumothorax requiring hospitalization but without any treatment breaks. He remains without evidence of disease at 11 month post-treatment. Other less common acute toxicities that were encountered included shortness of breath and grade 2 esophagitis in 3 patients (18%) and chest pain in 4 patients (25%) (Table 4). One patient required a treatment break of 21 days for treatment of arrhythmias and pneumonia that developed 2 days after the initiation of his boost treatment (same patient as the one mentioned above under treatment characteristics). A total of 4 (25%) patients including the one who reported hemoptysis developed grade 2 acute pneumonitis. All required steroid administration and one of the patients required hospitalization. There were no higher grade toxicities or any deaths resulting from the treatment.

**Table 4:** Toxicity profile of patients treated by SBRT boost

	
Fatigue	9 (56%)
Cough	8 (50%)
Shortness of Breath	3 (18.8%)
Chest pain	4 (25%)
Hemoptysis	1 (6%)
Esophagitis	3 (18.8%)
Pneumonitis	4 (25%)

## Discussion

Low survival outcomes have been reported for the treatment of locally advanced NSCLC with standard of care treatment of cCRT with locoregional control rates of approximately 65% with standard fractionation to 63 Gy [2]. Attempts to improve upon local disease control with radiation dose escalation have been undertaken [6,15,16] based on the radiobiologic principle that the larger the fraction cell kill, the greater the probability of disease control [17]. The RTOG 01–17 trial evaluated the feasibility of dose escalation concurrent with carboplatin and paclitaxel chemotherapy and identified a mean tolerated dose of 74 Gy [7]. On the basis of this result, the RTOG 06–17 trial randomized patients to 60 Gy versus 74 Gy, concurrent with platinum-based chemotherapy with or without cetuximab. Unfortunately, this trial closed early due to excess number of deaths on the high-dose arm in a futility analysis [8]. As the majority of locally advanced patients have larger-volume disease, it appears that the ability to dose escalate using conventional techniques concurrent with chemotherapy is hindered by the radiosensitive nature of the surrounding critical structures such as healthy lung, spinal cord, esophagus, heart, and brachial plexus [18]. Recent technological advances in conformal dose delivery have improved local control and complication rates. SBRT is particularly suitable for dose escalation because the rapid radiotherapy dose fall off with current image-guided radiotherapy technique. At a distance of 1.4 cm from the tumor, radiation dose fell rapidly from 174 Gy to 10 Gy [19].

A dosimetric analysis of CT data on locally advanced NSCLC patients undergoing cCRT to 50.4 Gy followed by SBRT boost to the tumor and involved nodes to either 16 Gy in 2 fractions or 28 Gy in 2 fractions showed dosimetric feasibility with respect to normal tissue tolerance [17]. Incorporation of positron emission tomography (PET) in SBRT treatment planning has also helped improve treatment accuracy, avoid marginal miss, and decrease treatment toxicity [20-22]. Dose escalation based on pre-treatment PET positivity and risk adapted treatment planning to maximally tolerated dose have resulted in excellent tumor control rates and minimal toxicity [22]. Using post-treatment PET at 5–6 weeks post-treatment to boost residual disease to higher dose has been successfully reported [21,23]. A recent prospective single institution study examined the safety and feasibility of using SBRT to boost PET residual disease for patients with mostly locally advanced NSCLC (31 out of 35 patients had stage III disease [21]. SBRT boost following cCRT was given in either 2 fractions of 10 Gy each or 3 fractions of 6.5 Gy each based on tumor location with local control rate of approximately 83% at a median followup of 13 months and 4 patients with acute radiation pneumonitis [21].

At a similar median followup our results show a median local control of 76% using a median SBRT boost dose of 25 Gy in 5 fractions also with acceptable toxicity. Our results also show acceptable regional and distant control rates. However, disease progression remains poor in our series. This could be due to the fact that there was no overlap between local, regional or distant control. Our sample size was underpowered to detect predictors of disease progression. However, advanced nodal disease was found to be a worse prognostic factor for progression in our series. This is consistent with published literature demonstrating poor prognostic association between mediastinal nodal involvement and disease progression in patients with Stage IIIA disease [24]. In our series age was also a worse prognostic outcome of both regional control and progression free survival. This is consistent with the recent analysis of seven RTOG trials that showed age to be an important predictor of loco-regional control and overall survival in patients with locally advanced lung cancer [25].

## Conclusion

Concurrent chemoradiation for locally advanced NSCLC remains associated with poor survival and significant toxicity. Our retrospective analysis shows that combination of cCRT to 50.4 Gy followed by SBRT boost to primary tumor and involved nodes using a risk adaptive regimen are possible with generally tolerable toxicity. Our data is limited by its retrospective nature, short follow-up, small sample size and heterogeneity of the patient population that are inherent in observational studies. Some of our patients had poor follow-up, and therefore disease recurrence may have been more common than reported. Clinical trials testing the role of using SBRT boost after definitive cCRT for locally advanced NSCLC are being undertaken [26] and results from these trials should provide a more authoritative answer to guide the treatment of this challenging disease.

## Competing interests

Actual or potential conflicts of interest do not exist for any of the authors.

## Authors’ contributions

SDK contributed to the conception, design, analysis, interpretation of the data, drafting of the manuscript, and critical revision of the manuscript for intellectual content. ZDH contributed to the data acquisition. HRL contributed to data acquisition, analysis and interpretation of the data. DM and DD contributed to data acquisition, analysis and interpretation of the data. NMN contributed to the conception, design, analysis, interpretation of the data, and revising the manuscript critically for important intellectual content. All authors read and approved the final manuscript.
